# Supplementary material for: Complexome profiling of the Chlamydomonas psb28 mutant reveals TEF5 as an early PSII assembly factor
Source: Plant Cell. 2025 Mar 17;37(6):koaf055. doi: 10.1093/plcell/koaf055 (PMC12164590; doi:10.1093/plcell/koaf055)
Supplement: koaf055_Supplementary_Data [file koaf055_supplementary_data.zip › psb28_interactive.zip!/plots/intensity/qualityPlots/peptides/LCI20_Cre06.g260450.t1.2.html]

### Cre06.g260450.t1.2

Trivial name: LCI20  
SeqCoverage [median]: 10.80  
Mass: 58.42 kDa  
  
 

| Sequence | MissedCleavage | Mass [Da] | wtIntensity | muIntensity | Score |
| --- | --- | --- | --- | --- | --- |
| AGGIFVPIIK | 0 | 1013.63 | 5899.82 | 22316.07 | 142.64 |
| IATLFVK | 0 | 790.50 | 1289.66 | 14812.48 | 114.02 |
| LEAMGPMSR | 0 | 990.46 | 0.00 | 385.83 | 85.73 |
| LSPPTITDTPDAPAAAAK | 0 | 1735.90 | 556.40 | 11536.18 | 184.31 |
| RLEAMGPMSR | 1 | 1146.56 | 410.21 | 4203.72 | 80.98 |
| SLAHSVGSEPGDDSR | 0 | 1512.68 | 2658.00 | 12211.90 | 68.94 |
|  |  |  | 10814.1 | 65466.2 |  |
